# Host Immune Response and Novel Diagnostic Approach to NTM Infections

**DOI:** 10.3390/ijms21124351

**Published:** 2020-06-18

**Authors:** Yuko Abe, Kiyoharu Fukushima, Yuki Hosono, Yuki Matsumoto, Daisuke Motooka, Naoko Ose, Shota Nakamura, Seigo Kitada, Hiroshi Kida, Atsushi Kumanogoh

**Affiliations:** 1 Department of Respiratory Medicine and Clinical Immunology, Osaka University Graduate School of Medicine, 2-2 Yamadaoka, Suita, Osaka 565-0871, Japan; y.abe@imed3.med.osaka-u.ac.jp (Y.A.); fukushima@imed3.med.osaka-u.ac.jp (K.F.); yk-hosono@hotmail.co.jp (Y.H.); kumanogo@imed3.med.osaka-u.ac.jp (A.K.); 2 Department of Respiratory Medicine, National Hospital Organization Osaka Toneyama Medical Centre, 5-1-1 Toneyama Toyonaka, Osaka 560-8552, Japan; 3 Department of Infection Metagenomics, Genome Information Research Center, Research Institute for Microbial Diseases (RIMD), Osaka University, 3-1 Yamadaoka, Suita, Osaka 565-0871, Japan; matsumoto@gen-info.osaka-u.ac.jp (Y.M.); daisukem@gen-info.osaka-u.ac.jp (D.M.); nshota@gen-info.osaka-u.ac.jp (S.N.); 4 Department of General Thoracic Surgery, Osaka University Graduate School of Medicine, 2-2 Yamadaoka, Suita, Osaka 565-0871, Japan; naokoose@thoracic.med.osaka-u.ac.jp; 5 Department of Respiratory Medicine, Yao Tokushukai General Hospital, 1-17 Wakakusa-cho, Yao, Osaka 581-0011, Japan; kitadas1@mac.com

**Keywords:** nontuberculous mycobacteria, host immune response, immunocompromised host, multilocus sequence typing database, GPL core IgA antibody

## Abstract

The incidence and prevalence of non-tuberculous mycobacteria (NTM) infections are steadily increasing worldwide, partially due to the increased incidence of immunocompromised conditions, such as the post-transplantation state. The importance of proper diagnosis and management of NTM infection has been recently recognized. Host immunological responses play integral roles in vulnerability to NTM infections, and may contribute to the onset of specific types of NTM infection. Furthermore, distinct NTM species are known to affect and attenuate these host immune responses in unique manners. Therefore, host immune responses must be understood with respect to each causative NTM species. Here, we review innate, cellular-mediated, and humoral immunity to NTM and provide perspectives on novel diagnostic approaches regarding each NTM species.

## 1. Introduction

**Figure 1:**
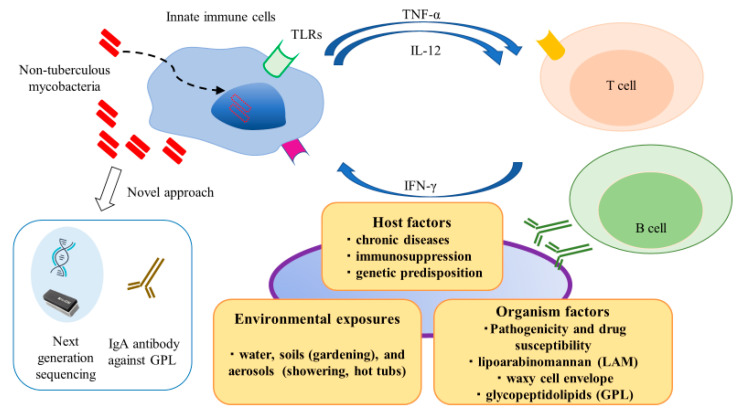
Schematic representation of complex interactions between hosts and pathogens in non-tuberculous mycobacteria (NTM) infection. Environmental exposures, host factors, and organismal factors contribute to development and progression of NTM infection. Comprehensive understanding of these processes is necessary for early and proper management of NTM infection.

Non-tuberculous mycobacteria (NTM) are ubiquitous environmental organisms of considerable clinical relevance, which are commonly found in water and soil [1]. Population-based data show that the incidence of NTM infections is increasing worldwide, notably, NTM infections have surpassed *Mycobacterium tuberculosis* (Mtb) infections in developed countries [2]. NTM infections occur in lymph nodes, skin and soft tissues, lung, and systemically (i.e., disseminated infection). Despite the ubiquitous presence of NTM species in the environment and presumably pervasive human exposure, the occurrence of NTM-related diseases is relatively infrequent [3]. This discrepancy suggests that NTM species possess low to moderate pathogenicity, such that host risk factors may play integral roles in vulnerability to NTM infections. Thus, individuals with abnormal immune systems exhibit an elevated risk of NTM infection; because of its chronic nature, NTM infection constitutes a significant health burden on various populations and is an important cause of morbidity and mortality [4,5]. As shown in Figure 1, environmental exposures, host factors, and organismal factors contribute to development and progression of NTM infection. Innate immune responses play crucial roles in recognizing and eliminating these pathogens. Furthermore, cytokine networks (e.g., tumor necrosis factor-α, interleukin [IL]-12, and interferon [IFN]-γ) play essential roles in regulating and bridging innate and adaptive immune responses through the induction and resolution of inflammation.

Although considerable information is available concerning human immune responses to mycobacteria, most of this information involves responses to Mtb. The Bacillus Calmette-Guérin (BCG) vaccine has been administered in several countries to prevent tuberculous meningitis in childhood, based on similarities in the immune reactions to Mtb and BCG. However, recent studies have shown that the human immune system exhibits some differences in responses to Mtb and NTM species, as well as responses to specific NTM subspecies. Therefore, an understanding of species and subspecies-specific human immune reaction is necessary to develop useful serodiagnostic tests and effective vaccines, as well as to discover new therapeutic targets in NTM. In this paper, we reviewed innate, cellular-mediated, and humoral immune responses to NTM infections and novel diagnostic approaches regarding each NTM species. We also reviewed reports that have focused on differences in immune responses to multiple subspecies of mycobacteria. Finally, we discussed what is needed in future studies regarding human immune responses to NTM.

## 2. Innate Immune Response to NTM Infection

The innate immune system is a form of host defense that promptly senses invading pathogens through pattern recognition receptors (PRRs). These receptors recognize molecular structures (i.e., pathogen-associated molecular patterns [PAMPs]) that are common to multiple pathogens. Major cell types in the innate immune system are macrophages and dendritic cells, which phagocytose and kill pathogens. These cells also produce inflammatory and anti-inflammatory cytokines through activation of multiple signaling pathways, triggered by PRR recognition of PAMPs. Mycobacterial PAMPs include components of the cell wall and nucleic acids. The mycobacterial cell wall is composed of lipids and polysaccharides. It also contains large quantities of mycolic acid (MA) [6,7]. Lipomannan (LM), lipoarabinomannan (LAM), phosphatidylinositol mannosides (PIMs), and MA are well-known specific components of the mycobacterial cell wall, which are reportedly ligands for PRRs [7]. LM and LAM are complex lipids on the mycobacterial cell surface and these lipids are presumed to be important in contact with the host [8,9]. The sugar moieties of those lipids differ among mycobacterial species. Additionally, nucleic acids from pathogens have been reported to serve as PAMPs, unmethylated CpG motifs of mycobacterial DNA are also recognized as PAMPs [10]. These mycobacterial PAMPs induce critical immune responses in innate immune cells through PRRs.

All PRRs sense mycobacteria and these sensing receptors include Toll-like receptors (TLRs), Nod-like receptors (NLRs), retinoic acid-inducible gene-I-like receptors, and C-type lectin receptors (CLRs). TLRs have fundamental roles in recognition of both intracellular and extracellular PAMPs [11]. In mucosal and innate immune cells, TLRs 1, 2, 4, 5, and 6 are expressed on the cell surface, while TLRs 3, 7, 8, and 9 are expressed on the intracellular endoplasmic reticulum. With the exception of TLR3, all TLRs transduce down-stream signals through myeloid differentiation factor 88 (MyD88) via TLR-MyD88 pathways [12]. Among these, TLR3 and TLR4 also have TLR-MyD88-independent pathway, and TLR domain-containing adapter inducing IFN-β (TRIF) is an adapter responsible for this pathway. On the other hand, TRIF can regulate TLR5 activity by inducing proteolytic degradation of TLR5 [13]. TLRs 2, 4, and 9 reportedly recognize mycobacteria [14,15,16,17]. TLR2 recognizes lipoproteins, peptidoglycan, and glycolipids (e.g., LAM, LM, and PIMs) [18,19]. With respect to NTM infection, TLR2 (not TLR4) has been reported to recognize *Mycobacterium avium* in vitro [20]. Glycopeptidolipids (GPLs), unique lipids in NTM, are reportedly recognized by TLR2 [21]. After infection with *M. avium*, TLR2-deficient mice showed suppression of bacterial clearance, compared with wild-type mice; conversely, TLR4-deficient mice showed similar bacterial clearance, compared with wild-type mice [22]. Feng et al. reported that bacterial clearance was suppressed more severely in MyD88-deficient mice than in TLR2-deficient mice. This finding indicated that MyD88-dependent signals leading to nuclear factor-κB (NF-κB) and MAPK activation via IL-1 receptor-associated kinase (IRAK) and TNF receptor-associated factor (TRAF) from multiple TLRs, including TLR2, might be necessary to regulate *M. avium* infection. In addition, TLR6- and TLR9-deficient mice were both found to exhibit impaired *M. avium* clearance [23,24]. In humans, impaired expression of TLR2 has been associated with NTM infection [25], specifically, patients with NTM infection showed lower TLR2, IL-12p40, and tumor necrosis factor-α mRNA levels in peripheral blood monocytes, compared with healthy controls [25].

Nod-like receptors (NLRs) are cytosolic PRRs, and essential component of multiprotein inflammasome complex [26]. Assembly and activation of inflammasomes are induced by oligomerization of NLRs upon their activation. Inflammasome activates the pro-caspase-1 to produce active caspase-1 subunits, which subsequently promotes the IL-1β maturation and secretion [27]. Inflammasome complex also play roles in response to NTM infection [28,29].

C-type lectin receptors (CLRs) constitute a family of receptors that bind sugar-containing ligands on the surface of pathogens. Several lipids on mycobacteria have been identified as ligands for CLRs. Trehalose-6,6-dimycolate, the mycobacterial cord factor, is the ligand of Macrophage-Inducible C-Type Lectin and Macrophage C-type Lectin [30]. Tri- and tetra-acylated PIMs are recognized by Dendritic Cell Immunoactivating Receptor [31]. Mannose-capped-LAM (Man-LAM), an abundant cell-wall lipoglycan, is known to be recognized by Dectin-2 [32]. Notably, Dectin-2-deficient mice showed inefficient *M. avium* clearance from the lung, compared with wild-type mice. Man-LAM induces both pro-inflammatory and anti-inflammatory cytokine production through Dectin-2, indicating that Dectin-2 might contribute to *M. avium* infection.

Other receptors have been reported to sense mycobacterial components. For example, mannose receptor C-types 1 and 2 recognize LAM and Man-LAM [33]. While specific intracellular adhesion molecule-3 grabbing molecule-3 senses LM and Man-LAM, dendritic cell-specific intracellular adhesion molecule-3-grabbing nonintegrin recognizes Man-LAM [34].

Macrophages play a central role in mycobacterial pathogenesis. Generally, macrophages eliminate mycobacteria through several mechanisms, such as production of reactive oxygen intermediates, reactive nitrogen intermediates, and free fatty acids [35]. However, some reports suggest that those mechanisms do not function properly in the context of *M. avium* infection [36]. Thus, *M. avium* may inhibit NF-κB activation and phagolysosome fusion [37]. Furthermore, *M. avium*-infected macrophages produce both pro-inflammatory cytokines and anti-inflammatory cytokines. Thus, several immunosuppression mechanisms can lead to NTM survival in hosts.

Several diseases, which cause defects in innate immunity, lead to enhanced susceptibility to NTM infections. Mutations in the *IKBA* or *IKBKG/NEMO* genes result in the inhibition of TLR-mediated NF-κB activation [38,39], which can lead to the onset of multiple infections, including NTM. Gene mutations, such as the Arg753Gln polymorphism in TLR2, are associated with tuberculosis [40].

## 3. Cellular-Mediated Immune Responses to NTM Infection

After pathogens have been sensed by the innate immune system, cytokine networks play crucial roles in bridging innate and adaptive immunity [41]. Among them, type I cytokines (e.g., IL-12 and IFN-γ) have been reported to be critical regulators of T cell responses in mycobacterial disease [42]. IL-12 represents the junction between innate and adaptative immunity [43]. Endocytosis of mycobacteria triggers IL-12 production by innate immune cells. CD4^+^ T cells, which are activated by IL-12, differentiate into T-helper 1 subpopulations (Th1). Th1 cells and CD8^+^ T cells, which are also activated by IL-12, secrete high amount of IFNγ which is extremely important for host defense against mycobacteria [44]. Therefore, the communication between innate and adaptive immunity, mediated by IFNγ and IL-12, plays a very important role in the control of infections by mycobacteria. In addition to Th1 T cells, Th17 T cells, another subpopulation of CD4^+^ T cells, produce IL17, IL21, and IL22 and affect the outcome of NTM infections by regulating neutrophil infiltration [45].

Mutations in five molecules in the IFN-γ/IL-12 pathway, including IFN-γ receptor 1 (IFN-γR1), IFN-γR2, IL-12p40, IL-12 receptor β1 (IL-12Rβ1), and STAT1, have been identified in a genetic syndrome with enhanced susceptibility to mycobacterial infection: Mendelian Susceptibility to Mycobacterial Disease [46]. STAT1 is a transcription factor downstream of the IFN-γ/IL-12 pathway. Various clinical syndromes with different severities have been reported in association with mutations of this gene.

The acquired immunodeficiency caused by production of anti-IFN-γ autoantibodies also leads to NTM infection [47]. Anti-IFN-γ autoantibodies are frequently found among Asian patients without acquired immune deficiency syndrome who exhibit disseminated NTM infection [48].

The loss of T-cell function is a risk factor for NTM infection. This includes severe combined immune deficiency [49], isolated CD4^+^ T lymphocyte deficiency [50], and acquired immunodeficiency syndrome induced by human immunodeficiency virus, which destroys CD4^+^ T lymphocytes [51].

Natural killer (NK) cells and natural killer T cells also play crucial roles in innate host defense against NTM infection. NK cells are granular lymphocytes with potent cytolytic capacity and they function during early infection and are independent of major histocompatibility complex signaling. MA, an abundant lipid component of the mycobacterial cell wall, is a ligand for the natural cytotoxicity receptor, NKp44, on NK cells [52]. NK cells produce IFN-γ and IL-22, which can inhibit intracellular mycobacterial growth by activating phagolysosomal fusion [53]. Natural killer T cells (NKT cells) are CD1-restricted T cells that possess semi-invariant T cell receptors. NKT cells recognize lipid antigens (e.g., MA, LAM, PIM, glucose monomycolate, and GroMN, a member of the CD1 family [54,55]) and can produce several cytokines immediately after activation [56]. NKT cells might play roles in modulating immune response after NTM infection, as well as Mtb infection [57].

### NTM Infections Affect Immunocompromised Organ Transplanted Patients

Solid organ transplant recipients, who require immunosuppressive agents to prevent rejection, are at greater risk for NTM infection due to depressed cellular-mediated immunity, compared with the general population. In contrast to the incidence of Mtb, which has decreased in developed countries [58], NTM infections now represent a considerable disease burden in organ transplant recipients. Moreover, NTM infections cause substantial morbidity and mortality, because of difficulties in disease recognition, diagnosis, and complex drug interactions [59]. In organ transplant recipients, the median onset of NTM infection typically occurs > 1 year after transplantation [60]. NTM infection is reported to occur in 0.5–8.0% of patients who undergo lung transplantation [60,61]. Pleuropulmonary disease is the most common manifestation of NTM infection after transplantation, occurring in > 50% of patients; cutaneous and disseminated infections are also common in these patients [61]. Among NTM species, *M. avium* complex (MAC), *Mycobacterium kansasii*, *Mycobacterium abscessus*, and *Mycobacterium xenopi* are frequently isolated [61]. Prednisolone and calcineurin inhibitors (e.g., cyclosporin) are the main immunosuppressive drugs after transplantation. Because these drugs are metabolized by CYP450 enzyme, which is induced by rifampicin and reduced by macrolides, drug interactions associated with CYP450 must be considered when adjusting the dosages of these drugs, which is often difficult.

In a previous study, 71 patients received lung transplantation between January 2000 and December 2019 in our hospital, of these 71 patients, four (5.6%) met diagnostic criteria for NTM pulmonary disease, based on American Thoracic Society and Infectious Diseases Society of America guidelines, and received treatment. NTM species were not detected in any examination prior to transplantation. Surveillance bronchoscopy examinations were performed several times during the first year after transplantation, then annually thereafter, in accordance with our institutional protocol [62]. After the fifth year, clinically indicated bronchoscopy examinations were performed, in addition to regular sputum and imaging examinations, when rejection or respiratory infection was suspected. As shown in Table 1, all patients exhibited pleuropulmonary disease. Two (50%) patients died after treatment of NTM. In Patient 2, *M. abscessus* infection after transplantation caused lung destruction, which resulted in exacerbation of chronic respiratory failure. In Patient 4, *M. intracellulare* infection occurred 1 year after transplantation; despite extensive multidrug treatment with rifampicin, ethambutol, and clarithromycin, the patient exhibited acute humoral injection and died. In our case series, the time of onset and incidence of NTM infection after lung transplantation were comparable to the findings in previous reports. Several studies have shown that MAC and *M. abscessus* are the most common pathogens in lung transplantation recipients [61,63], which are similar to our findings. Mortality is higher in post-transplant NTM infections, thus, early diagnosis and treatment are extremely important. As in our Patient 4, drug-drug interactions create difficulty in maintaining balance between immunosuppressive drugs and antimycobacterial therapy in organ transplant recipients with NTM infections. In our study, all patients exhibited non-cavitary nodular bronchiectatic disease, which results from airway clearance defects. Thus, the weakened immune response would have led to poor pathogen clearance. Additionally, the findings suggest a close relationship between cell-mediated immunity and cavity formation [64]. Overall, prevention of NTM infection among post-transplant patients is necessary by performing serodiagnostic detection of early-stage NTM infection, as well latent infection, in both donors and recipients. Furthermore, NTM must be identified at the subspecies level by genomic analysis, which enables treatment with NTM-subspecies-specific regimens.

## 4. Humoral Immune Response to NTM Infection

Mycobacteria are intracellular pathogens; thus, research concerning human protective immunity against these bacteria has mainly focused on cell-mediated immunity. However, B cells or antibody-responsive innate immune cells, which bear Fc receptors, are the major cellular components of granulomatous tissue in lungs affected by mycobacterial disease [65]. Furthermore, experimental animal models of mycobacterial infection have revealed that humoral immunity plays a role in the response to this type of infection [66]. Blood-based transcriptomic analysis among human immunodeficiency virus-infected patients revealed that complement activation via immune complex formation is an early event in tuberculosis [67]. Thus, a better understanding of humoral immunity against mycobacteria is necessary for the development of effective treatments and vaccines [68]. Mycobacteria are known to have cell wall-bound, cytoplasmic antigens and they also actively secrete immunogenic proteins. Thus far, multiple serum antibodies against these mycobacterial antigens have been mainly studied for use as biomarkers [69]. The possibility of protective antibodies against mycobacteria was demonstrated by an inverse relationship between the presence of the antibody and susceptibility to infection and disease in several vaccine studies [68].

To clarify the role of humoral immunity in mycobacterial disease, monoclonal antibodies against mycobacterial antigens have been produced and analyzed. Hamasur et al. showed that a mouse monoclonal IgG1 antibody to lipoarabinomannan (SMITB14) was protective against tuberculosis in intravenously infected mice [70]. Balu et al. produced a recombinant human IgA1 antibody against mycobacterial α-crystallin (2E9IgA1) and showed its protective effect from intranasally infected mycobacteria in human CD89 (Fcα R1) transgenic mice [71].

Factors that affect antibody function have been investigated. The Fc class-switch recombination, which results in section of different isotypes, affects antibody distribution. Zimmermann et al. showed that IgA (not IgG) antibodies to LAM and heparin-binding hemagglutinin blocked Mtb activity, suggesting the importance of mucosal immunity [72]. Posttranslational modification by glycosylation is another method to modulate antibody function. Lu et al. found that the constant (Fc) antibody domain is differentially glycosylated according to the state of tuberculosis disease. Di-galactosylation or sialylation of asparagine residue (Asp297) in the CH2 domain was more frequently found in antibodies from patients with latent tuberculosis and associated with enhanced Fc effector profiles (including selective binding to FcγRIII), compared with patients with active tuberculosis [73].

In the pathology of pulmonary mycobacterial disease, the early stage of Mtb infection in the lung is a necrotizing bronchopneumonia, followed by the formation of granuloma with caseous necrosis. It is characterized by a central zone of necrosis surrounded by epithelioid histiocytes. These pathological features are also found in NTM infections [74]. Although the histological features of Mtb and NTM infections are usually very similar, biopsies from immunocompromised individuals such as HIV/AIDS patients frequently show variant patterns of ill-defined granulomas, distributed randomly, so called miliary disease, more common in Mtb than NTM [75]. As histological and radiological findings consistent with bronchiectasis and centrilobular nodules, infiltration of lymphocytes and epithelioid cells under the bronchiolar mucosa is widely observed in Mtb and NTM infections, and these findings are considered to be the reflection of on-going pathologic process of bronchiolitis and bronchiectasis [74,75]. Future studies evaluating the differences in pathological findings between Mtb and NTM are necessary.

Cavity formation is the hallmark of active mycobacterial disease. Delayed type of hypersensitivity to mycobacterial antigens is known as an essential factor for caseous necrosis and cavity formation [64]. Comprehensive analysis of serum antibodies using an entire Mtb proteome microarray suggested that the number of mycobacterial antigens—including those with adjuvant activity for cellular-mediated immunity, which were recognized by patients’ serum antibodies—increased with an increasing bacterial burden [76]. These antibodies might mediate anti-inflammatory functions through sequestration of mycobacteria-derived adjuvants and prevention of cavity formation.

## 5. Novel Diagnostic Approach to NTM Infections

### 5.1. Serodiagnosis for Mycobacterium Avium Complex Pulmonary Disease 

Several NTM species, including MAC, *M abscessus*, and *M chelonae*, express GPLs in the outer layer of the mycobacterial envelope. The core structure of GPLs, which is common to all GPLs, is a 3-hydroxy or 3-methoxy C26–C33 fatty acyl chain N-linked to a tripeptide-amino-alcohol, D-phenylalanine-D-*allo*-threonine-D-alanine-L-alaninol. In MAC, the *allo*-threonine of this GPL-core is glycosylated by different oligosaccharide-chains, thus producing 31 serotype-specific GPLs. Each MAC strain expresses one of these serotype-specific GPLs [77]. A serological test kit was previously developed for use in diagnosis of MAC disease. The kit was commercialized (Capilia™ MAC Ab ELISA, Tauns Co., Ltd., Shizuoka, Japan) and has been widely used in clinical practice in Japan since 2011 [78]. The kit measures serum IgA antibody against the GPL core, which is a major surface antigen of MAC. Antibody levels are elevated in patients with MAC pulmonary disease, but not in patients with other pulmonary infections (e.g., pulmonary tuberculosis) or in individuals with contamination or colonization alone. A meta-analysis reported that respective estimated sensitivity and specificity values were 69.6% (95% confidence interval, 62.1–76.1%) and 90.6% (95% confidence interval, 83.6–95.1%) [79]. Moreover, respective positive and negative likelihood ratios were 7.4 (95% confidence interval, 4.1–13.8) and 0.34 (95% confidence interval, 0.26–0.43). Thus, the kit is useful for simple and rapid diagnosis of MAC pulmonary disease.

Subsequent studies revealed that antibody levels were significantly reduced by successful multidrug combination chemotherapy, whereas the levels increased in patients with recurrence [80]. In addition, antibody levels decreased after surgery in most patients who underwent surgical resection, compared with the levels before surgery [81]. These results suggested that the antibody levels reflect disease activity; serial measurement of antibody levels in a patient may be useful for monitoring disease activity.

In patients with tuberculosis, the establishment of a host adaptive immune response toward Mtb, which is now measured by an interferon-γ release assay, is regarded as a sign of latent infection. It should be clarified whether a condition similar to latent tuberculosis infection also exists in patients with NTM infection, and whether positive results in the Capilia™ MAC assay are indicative of this condition.

In summary, serodiagnosis has good sensitivity, specificity, and positive predictive value. Combined with current diagnostic criteria, serodiagnosis is useful as an auxiliary diagnostic tool and may be useful for monitoring disease activity in a patient. However, there is insufficient information to endorse its routine use in clinical practice worldwide. Most published data have been from East Asia, thus, further validations are needed in various regions.

### 5.2. Rapid and Comprehensive Identification of NTM Species by MLST Database 

NTM Infections are extremely diverse and manifest as a large spectrum of diseases that affect several organs and immune responses to these pathogens differ depending on the species [82]. Furthermore, MAC, one of the most frequent pathogens among 200 NTM species, consists of 31 distinct serotypes, which are distinguished by serotype-specific GPLs in the outer layer of the mycobacterial envelope. Each serotype of MAC is recognized differentially by the host immune system and affects the clinical course or prognosis [83]. Recently, differences in pathogenicity among NTM subspecies has begun to receive attention. Although the prevalence of NTM infection is lower than that of Mtb infection, more accurate identification is necessary for NTM strains that exhibit a wide variety of genetic backgrounds, such as virulence factors and antimicrobial resistance. In this context, various methods have been proposed thus far for identification of mycobacterial species. In addition, an understanding of subspecies-specific human immune reactions is necessary to develop useful serodiagnostic tests and effective vaccines, as well as to discover new therapeutic targets in NTM.

Currently, next-generation sequencers enable rapid and precise identification of NTM subspecies by multi-locus sequence typing (MLST). This method has facilitated accurate identification of important subspecies during treatment of NTM infection. Accumulation and integrated comparison of genomic sequences obtained by MLST may enable more detailed typing related to prognosis and treatment resistance among new subspecies.

Typical molecular diagnostic techniques utilize genetic information for identification of NTM species [84]. Polymerase chain reaction and transcription reverse-transcription concerted reaction assays are used in practice to detect several housekeeping genes (e.g., *16SrRNA*, *rpoB*, and *hsp65*). Although these conventional amplification-dependent analyses are highly sensitive and can directly identify clinical specimens without the requirement for culturing, detectable species remain limited to well-known mycobacterial species. MLST is also a well-known method that utilizes genetic information for accurate species identification. The MLST approach achieves strain-level identification by preparing a corresponding MLST database consisting of profiles from the taxon of interest [85]. A widely used MLST approach can discriminate organisms at the species level by analysis of seven housekeeping genes commonly conserved among taxa. There are other approaches for identification of bacteria without the use of genetic information, such as mass spectrometry [86] or microspectrometry [87]. The mass spectrometry-based method is a low-cost, well-established approach for mycobacterial identification and rapid detection of pathogens from isolates at the species level. However, detectable species are limited, as mentioned for the polymerase chain reaction- and transcription reverse-transcription concerted-based methods. For species identification, the genetic information-based approach has the highest specificity because current taxonomic classification relies on genomic aspects due to the development of molecular diagnostic techniques.

In recent decades, novel approaches for using large-scale genetic information obtained from whole-genome sequencing are coming to the forefront due to the development of next-generation sequencing (NGS) technologies. The whole-genome sequencing approach can directly discriminate species at the strain level by focusing on species-specific regions [88,89], thus enabling specific detection of pathogens with a small amount of data (e.g., from metagenomic DNA analyses). Other identification methods also utilize the overall genome relatedness index [90], such as average nucleotide identity [91,92] or genome-to-genome distance [93]. Overall genome relatedness index approaches using whole-genome information are highly sensitive and applicable to many organisms with available genome sequences. The average nucleotide identity index has been used as a measure of taxonomy classification. This index is also employed for mycobacterial species identification. However, the average nucleotide identity index cannot distinguish two genetically closely related but different species, notably, various concepts of species (e.g., biological, ecological, and genomic aspects) coexist in the taxonomy classification [15,94]. For mycobacterial identification, large-scale extensions of the MLST approach, such as PubMLST [95] or mlstverse [96], are used because of the requirement of subspecies level identification. PubMLST is a widely used MLST approach, consisting of conventional MLST and ribosomal MLST for bacterial identification. In the ribosomal MLST approach, PubMLST utilizes 53 ribosomal genes, thereby covering a variety of species. In contrast, mlstverse uses a variable number of genes per profile and external MLST databases can be selected. In mlstverse, 184 genes associated with species-specific pathogenesis and drug resistance, as well as 53 ribosomal genes, were integrated into profiles for mycobacterial identification. A large-scale MLST approach is applicable to > 180 mycobacterial species with both high sensitivity and specificity, based on core and accessory gene sequences, however, it currently requires preparation of culture isolates.

**Figure 2:**
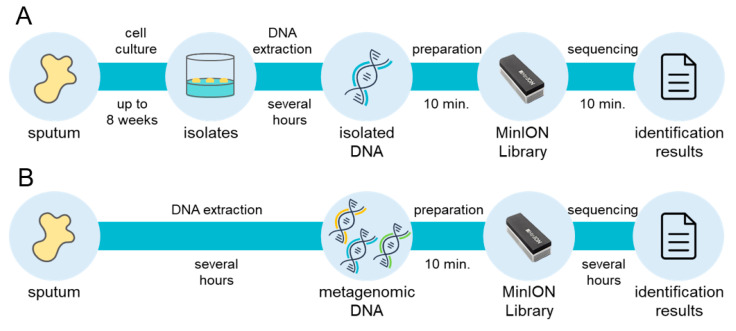
Rapid and comprehensive identification of mycobacteria. Large-scale extension of multi-locus sequence typing (MLST) approach currently achieves comprehensive identification of mycobacteria within 20 min from isolated DNA, although up to 8 weeks is needed for the cell culture step (**A**). A direct metagenomic approach will remove the bottleneck and enable rapid and comprehensive detection of mycobacteria (**B**).

From a clinical perspective, the mycobacterial incubation time is a bottleneck in the overall diagnostic process, because up to 8 weeks of growth may be needed to obtain culture isolates of slow-growing species. With the MLST approach using comprehensive genomic information regarding mycobacteria, the Oxford Nanopore Technologies MinION sequencer enables diagnosis within 20 min from isolated genomic DNA with mlstverse [96] (Figure 2). In addition, NGS technologies may allow direct detection of pathogens from metagenomic DNA because they present sequencing results in real-time. A recent study proposed a direct Mtb detection method using MinION [97]. Further comprehensive and highly sensitive identification methods to discriminate mycobacteria are necessary because NTM species should be distinguished from Mtb. Accurate and rapid identification of the entire Mycobacteriaceae family may become possible with the use of NGS (Figure 2). This whole-genomic approach will facilitate appropriate treatment plans for NTM infection with reductions in mycobacteria-related deaths worldwide.

## 6. Conclusion and Future Direction 

NTM infections are increasing worldwide, but there remains a considerable lack of clarity regarding their mechanisms, compared to Mtb infection. Immunocompromised hosts are more susceptible to NTM infection, compared to the general population. Therefore, host immune responses to NTM are important, indeed, chronic disease and treatments with immunosuppressive drugs increase the morbidity and mortality of NTM infections. Both innate and acquired immune responses—mediated by various types of immune cells, cytokines, and autoantibodies—play important roles in modulating immune responses to NTM pathogenesis.

The type of disease, fibrocavitary or nodular-bronchiectatic, is presumably determined by both host- and pathogen-factors, which affect disease course. Further research is needed regarding more comprehensive human immune responses to NTM, depending on the subspecies levels. Identification of these interactions between various pathogens and the host may lead to development of vaccines and new drugs for treatment of NTM infections. Indeed, with the advancement of immunotherapy, lots of discussions are happening to “boost” a weakened immune system through targeted augmentation of immune responses by adjuvant immune therapies [98]. For example, induction of strong T-cell mediated immunity have been reported by the use of vaccines using antigen-presenting cells and B cells [99]. Such immune modulating interventions would be promising and applicable for reducing the global burden of NTM disease.

## Figures and Tables

**Table 1:** Clinical characteristics and outcomes of patients with NTM infection after lung transplantation.

No.	Age	Sex	Primary Disease	Procedure of Transplant	Immuno-Suppressive Agents	Species	Radiologic Features	Site of Infection	Time to Isolate (months)	Treatment	Outcome
1	10	M	PAH	DLT	TAC, MMF, PSL	*M. gordonae*	Non-cavitary NB	Transplanted	76	None	Alive
2	35	F	BE	DLT	CyA, MMF, PSL	*M. abscessus*	Non-cavitary NB	Transplanted	82	IMP, CAM, AMK	Dead
3	41	F	LAM	SLT	CyA, MMF, PSL	*M. abscessus*	Non-cavitary NB	Transplanted	58	IPM, AMK, AZM	Alive
4	39	M	IIP	SLT	CyA, MMF, PSL	*M. intracellulare*	Non-cavitary NB	Native	12	RFP, EB, CAM	Dead

PAH: pulmonary arterial hypertension, NB: nodular bronchiectatic, BE: bronchiectasis, LAM: lymphangioleiomyomatosis, IIP: idiopathic interstitial pneumonia, DLT: double lung transplantation, SLT: single lung transplantation, TAC: tacrolimus, MMF: mycophenolate mofetil, PSL: prednisolone, CyA: cyclosporine, AZM: azithromycin, IPM: imipenem/cilastatin, AMK: amikacin, RFP: rifampicin, EB: ethambutol, and CAM: clarithromycin.

## References

[1] Falkinham J.O. (2013). Ecology of Nontuberculous Mycobacteria—Where Do Human Infections Come from?. Semin. Respir. Crit. Care Med..

[2] Jennifer A., Timothy B.F., Yihe G.D., Jennifer R.H., Kenneth N.O., Adrian Z., Stacey H.D., Rebecca P. (2017). Epidemiology of Nontuberculous Mycobacterial Lung Disease and Tuberculosis, Hawaii, USA. Emerg. Infect. Dis..

[3] Honda J.R., Alper S., Bai X., Chan E.D. (2018). Acquired and genetic host susceptibility factors and microbial pathogenic factors that predispose to nontuberculous mycobacterial infections. Curr. Opin. Immunol..

[4] Gochi M., Takayanagi N., Kanauchi T., Ishiguro T., Yanagisawa T., Sugita Y. (2015). Retrospective study of the predictors of mortality and radiographic deterioration in 782 patients with nodular/bronchiectatic Mycobacterium avium complex lung disease. BMJ Open..

[5] Fukushima K., Kitada S., Abe Y., Yamamoto Y., Matsuki T., Kagawa H., Oshitani Y., Tsujino K., Yoshimura K., Miki M. (2020). Long-term treatment outcome of progressive mycobacterium avium complex pulmonary disease. J. Clin. Med..

[6] Syal K., Maiti K., Naresh K., Avaji P.G., Chatterji D., Jayaraman N. (2016). Synthetic arabinomannan glycolipids impede mycobacterial growth, sliding motility and biofilm structure. Glycoconj. J..

[7] Dulberger C.L., Rubin E.J., Boutte C.C. (2020). The mycobacterial cell envelope – A moving target. Nat. Rev. Microbiol..

[8] Van de Weerd R., Boot M., Maaskant J., Sparrius M., Verboom T., van Leeuwen L.M., Burggraaf M.J., Paauw N.J., Dainese E., Manganelli R. (2016). Inorganic Phosphate Limitation Modulates Capsular Polysaccharide Composition in Mycobacteria. J. Biol. Chem..

[9] Dufrisne B.M., Jorge C.D., Timoteo C.G., Petrou V.I., Ashraf K.U., Banerjee S., Clarke O.B., Santos H., Mancia F. (2020). Structural and Functional Characterization of Phosphatidylinositol-Phosphate Biosynthesis in Mycobacteria. J. Mol. Biol..

[10] Tokunaga T., Yamamoto T., Yamamoto S. (1999). How bcg led to the discovery of immunostimulatory DNA. Jpn. J. Infect. Dis..

[11] Fitzgerald K.A., Kagan J.C. (2020). Toll-like Receptors and the Control of Immunity. Cell..

[12] Uematsu S., Akira S. (2007). Toll-like receptors and Type I interferons. J. Biol. Chem..

[13] Choi Y.J., Im E., Pothoulakis C., Rhee S.H. (2010). TRIF modulates TLR5-dependent responses by inducing proteolytic degradation of TLR5. J. Biol. Chem..

[14] Takeuchi O., Akira S. (2010). Pattern recognition receptors and inflammation. Cell.

[15] Vu A., Calzadilla A., Gidfar S., Calderon-Candelario R., Mirsaeidi M. (2017). Toll-like receptors in mycobacterial infection. Eur. J. Pharmacol..

[16] Mortaz E., Adcock I.M., Tabarsi P., Masjedi M.R., Mansouri D., Velayati A.A., Casanova J.L., Barnes P.J. (2015). Interaction of Pattern Recognition Receptors with Mycobacterium Tuberculosis. J. Clin. Immunol..

[17] Kurt-Jones E.A., Popova L., Kwinn L., Haynes L.M., Jones L.P., Tripp R.A., Walsh E.E., Freeman M.W., Golenbock D.T., Anderson L.J. (2000). Pattern recognition receptors tlr4 and cd14 mediate response to respiratory syncytial virus. Nat. Immunol..

[18] Saraav I., Singh S., Sharma S. (2014). Outcome of Mycobacterium tuberculosis and Toll-like receptor interaction: Immune response or immune evasion?. Immunol. Cell Biol..

[19] Shukla S., Richardson E.T., Drage M.G., Boom W.H., Harding C.V. (2018). Mycobacterium tuberculosis Lipoprotein and Lipoglycan Binding to Toll-Like Receptor 2 Correlates with Agonist Activity and Functional Outcomes. Infect. Immun..

[20] Lien E., Sellati T.J., Yoshimura A., Flo T.H., Rawadi G., Finberg R.W., Carroll J.D., Espevik T., Ingalls R.R., Radolf J.D. (1999). Toll-like receptor 2 functions as a pattern recognition receptor for diverse bacterial products. J. Biol. Chem..

[21] Sweet L., Schorey J.S. (2006). Glycopeptidolipids from mycobacterium avium promote macrophage activation in a tlr2- and myd88-dependent manner. J. Leukoc. Biol..

[22] Feng C.G., Scanga C.A., Collazo-Custodio C.M., Cheever A.W., Hieny S., Caspar P., Sher A. (2003). Mice lacking myeloid differentiation factor 88 display profound defects in host resistance and immune responses to mycobacterium avium infection not exhibited by toll-like receptor 2 (tlr2)- and tlr4-deficient animals. J. Immunol..

[23] Marinho F.A., de Paula R.R., Mendes A.C., de Almeida L.A., Gomes M.T., Carvalho N.B., Oliveira F.S., Caliari M.V., Oliveira S.C. (2013). Toll-like receptor 6 senses mycobacterium avium and is required for efficient control of mycobacterial infection. Eur. J. Immunol..

[24] Carvalho N.B., Oliveira F.S., Duraes F.V., de Almeida L.A., Florido M., Prata L.O., Caliari M.V., Appelberg R., Oliveira S.C. (2011). Toll-like receptor 9 is required for full host resistance to mycobacterium avium infection but plays no role in induction of th1 responses. Infect. Immun..

[25] Ryu Y.J., Kim E.J., Lee S.H., Kim S.Y., Suh G.Y., Chung M.P., Kim H., Kwon O.J., Koh W.J. (2007). Impaired expression of toll-like receptor 2 in nontuberculous mycobacterial lung disease. Eur. Respir. J..

[26] Meylan E., Tschopp J., Karin M. (2006). Intracellular pattern recognition receptors in the host response. Nature.

[27] van de Veerdonk F.L., Netea M.G., Dinarello C.A., Joosten L.A. (2011). Inflammasome activation and IL-1beta and IL-18 processing during infection. Trends Immunol..

[28] Wu M.F., Shu C.C., Wang J.Y., Yan B.S., Lai H.C., Chiang B.L., Wu L.S., Yu C.J. (2019). NLRP3 inflammasome is attenuated in patients with Mycobacterium avium complex lung disease and correlated with decreased interleukin-1beta response and host susceptibility. Sci. Rep..

[29] Lee H.M., Yuk J.M., Kim K.H., Jang J., Kang G., Park J.B., Son J.W., Jo E.K. (2012). Mycobacterium abscessus activates the NLRP3 inflammasome via Dectin-1-Syk and p62/SQSTM1. Immunol. Cell Biol..

[30] Miyake Y., Toyonaga K., Mori D., Kakuta S., Hoshino Y., Oyamada A., Yamada H., Ono K., Suyama M., Iwakura Y. (2013). C-type lectin mcl is an fcrgamma-coupled receptor that mediates the adjuvanticity of mycobacterial cord factor. Immunity.

[31] Toyonaga K., Torigoe S., Motomura Y., Kamichi T., Hayashi J.M., Morita Y.S., Noguchi N., Chuma Y., Kiyohara H., Matsuo K. (2016). C-type lectin receptor dcar recognizes mycobacterial phosphatidyl-inositol mannosides to promote a th1 response during infection. Immunity.

[32] Yonekawa A., Saijo S., Hoshino Y., Miyake Y., Ishikawa E., Suzukawa M., Inoue H., Tanaka M., Yoneyama M., Oh-Hora M. (2014). Dectin-2 is a direct receptor for mannose-capped lipoarabinomannan of mycobacteria. Immunity.

[33] Kang P.B., Azad A.K., Torrelles J.B., Kaufman T.M., Beharka A., Tibesar E., DesJardin L.E., Schlesinger L.S. (2005). The human macrophage mannose receptor directs mycobacterium tuberculosis lipoarabinomannan-mediated phagosome biogenesis. J. Exp. Med..

[34] Geijtenbeek T.B., Van Vliet S.J., Koppel E.A., Sanchez-Hernandez M., Vandenbroucke-Grauls C.M., Appelmelk B., Van Kooyk Y. (2003). Mycobacteria target dc-sign to suppress dendritic cell function. J. Exp. Med..

[35] Akaki T., Sato K., Shimizu T., Sano C., Kajitani H., Dekio S., Tomioka H. (1997). Effector molecules in expression of the antimicrobial activity of macrophages against mycobacterium avium complex: Roles of reactive nitrogen intermediates, reactive oxygen intermediates, and free fatty acids. J. Leukoc. Biol..

[36] Gomes M.S., Florido M., Pais T.F., Appelberg R. (1999). Improved clearance of mycobacterium avium upon disruption of the inducible nitric oxide synthase gene. J. Immunol..

[37] Gutierrez M.G., Mishra B.B., Jordao L., Elliott E., Anes E., Griffiths G. (2008). Nf-kappa b activation controls phagolysosome fusion-mediated killing of mycobacteria by macrophages. J. Immunol..

[38] Sexton P., Harrison A.C. (2008). Susceptibility to nontuberculous mycobacterial lung disease. Eur. Respir. J..

[39] Doffinger R., Smahi A., Bessia C., Geissmann F., Feinberg J., Durandy A., Bodemer C., Kenwrick S., Dupuis-Girod S., Blanche S. (2001). X-linked anhidrotic ectodermal dysplasia with immunodeficiency is caused by impaired nf-kappab signaling. Nat. Genet..

[40] Ogus A.C., Yoldas B., Ozdemir T., Uguz A., Olcen S., Keser I., Coskun M., Cilli A., Yegin O. (2004). The arg753gln polymorphism of the human toll-like receptor 2 gene in tuberculosis disease. Eur. Respir. J..

[41] Lindestam Arlehamn C.S., Lewinsohn D., Sette A., Lewinsohn D. (2014). Antigens for CD4 and CD8 T cells in tuberculosis. Cold Spring Harb. Perspect. Med..

[42] Ramirez-Alejo N., Santos-Argumedo L. (2014). Innate defects of the il-12/ifn-gamma axis in susceptibility to infections by mycobacteria and salmonella. J. Interferon Cytokine Res..

[43] Kaufmann S.H. (2002). Protection against tuberculosis: Cytokines, T cells, and macrophages. Ann. Rheum. Dis..

[44] Serbina N.V., Flynn J.L. (2001). CD8(+) T cells participate in the memory immune response to Mycobacterium tuberculosis. Infect. Immun..

[45] Matsuyama M., Ishii Y., Yageta Y., Ohtsuka S., Ano S., Matsuno Y., Morishima Y., Yoh K., Takahashi S., Ogawa K. (2014). Role of Th1/Th17 balance regulated by T-bet in a mouse model of Mycobacterium avium complex disease. J. Immunol..

[46] Al-Herz W., Bousfiha A., Casanova J.L., Chatila T., Conley M.E., Cunningham-Rundles C., Etzioni A., Franco J.L., Gaspar H.B., Holland S.M. (2014). Primary immunodeficiency diseases: An update on the classification from the international union of immunological societies expert committee for primary immunodeficiency. Front. Immunol..

[47] Otome O., O’Reilly M., Lim L. (2015). Disseminated mycobacterium haemophilum skeletal disease in a patient with interferon-gamma deficiency. Intern. Med. J..

[48] Valour F., Perpoint T., Senechal A., Kong X.F., Bustamante J., Ferry T., Chidiac C., Ader F., Lyon T. (2016). Interferon-gamma Autoantibodies as Predisposing Factor for Nontuberculous Mycobacterial Infection. Emerg. Infect. Dis..

[49] Marciano B.E., Huang C.Y., Joshi G., Rezaei N., Carvalho B.C., Allwood Z., Ikinciogullari A., Reda S.M., Gennery A., Thon V. (2014). Bcg vaccination in patients with severe combined immunodeficiency: Complications, risks, and vaccination policies. J. Allergy Clin. Immunol..

[50] Yarmohammadi H., Cunningham-Rundles C. (2017). Idiopathic CD4 lymphocytopenia: Pathogenesis, etiologies, clinical presentations and treatment strategies. Ann. Allergy Asthma Immunol..

[51] Hariadi N.I., Blackwood R.A. (2017). Disseminated Mycobacterium Avium Complex in an Adolescent with Perinatally-Acquired HIV Infection. Infect. Dis. Rep..

[52] Parodi M., Favoreel H., Candiano G., Gaggero S., Sivori S., Mingari M.C., Moretta L., Vitale M., Cantoni C. (2019). NKp44-NKp44 Ligand Interactions in the Regulation of Natural Killer Cells and Other Innate Lymphoid Cells in Humans. Front. Immunol..

[53] Rocco J.M., Irani V.R. (2011). Mycobacterium avium and modulation of the host macrophage immune mechanisms. Int. J. Tuberc. Lung Dis..

[54] James C.A., Seshadri C. (2020). T Cell Responses to Mycobacterial Glycolipids: On the Spectrum of "Innateness". Front. Immunol..

[55] Layre E., Collmann A., Bastian M., Mariotti S., Czaplicki J., Prandi J., Mori L., Stenger S., De Libero G., Puzo G. (2009). Mycolic acids constitute a scaffold for mycobacterial lipid antigens stimulating cd1-restricted t cells. Chem. Biol..

[56] Kinjo Y., Takatsuka S., Kitano N., Kawakubo S., Abe M., Ueno K., Miyazaki Y. (2018). Functions of CD1d-Restricted Invariant Natural Killer T Cells in Antimicrobial Immunity and Potential Applications for Infection Control. Front. Immunol..

[57] Kwon B.E., Ahn J.H., Park E.K., Jeong H., Lee H.J., Jung Y.J., Shin S.J., Jeong H.S., Yoo J.S., Shin E. (2019). B Cell-Based Vaccine Transduced With ESAT6-Expressing Vaccinia Virus and Presenting alpha-Galactosylceramide Is a Novel Vaccine Candidate Against ESAT6-Expressing Mycobacterial Diseases. Front. Immunol..

[58] Hagiya H., Koyama T., Zamami Y., Minato Y., Tatebe Y., Mikami N., Teratani Y., Ohshima A., Shinomiya K., Kitamura Y. (2018). Trends in incidence and mortality of tuberculosis in japan: A population-based study, 1997–2016. Epidemiol. Infect..

[59] Daley C.L. (2009). Nontuberculous mycobacterial disease in transplant recipients: Early diagnosis and treatment. Curr. Opin. Organ. Transplant..

[60] Longworth S.A., Daly J.S. (2019). Management of infections due to nontuberculous mycobacteria in solid organ transplant recipients-guidelines from the American society of transplantation infectious diseases community of practice. Clin. Transplant..

[61] Malouf M.A., Glanville A.R. (1999). The spectrum of mycobacterial infection after lung transplantation. Am. J. Respir. Crit. Care Med..

[62] Ose N., Minami M., Funaki S., Kanou T., Kanzaki R., Shintani Y. (2019). Nontuberculous mycobacterial infection after lung transplantation: A report of four cases. Surg. Case Rep..

[63] Shah S.K., McAnally K.J., Seoane L., Lombard G.A., LaPlace S.G., Lick S., Dhillon G.S., Valentine V.G. (2016). Analysis of pulmonary non-tuberculous mycobacterial infections after lung transplantation. Transpl. Infect. Dis..

[64] Yamamura Y., Maeda H., Ogawa Y., Hashimoto T. (1986). Experimental pulmonary cavity formation by mycobacterial components and synthetic adjuvants. Microbiol. Immunol..

[65] Tsai M.C., Chakravarty S., Zhu G., Xu J., Tanaka K., Koch C., Tufariello J., Flynn J., Chan J. (2006). Characterization of the tuberculous granuloma in murine and human lungs: Cellular composition and relative tissue oxygen tension. Cell Microbiol..

[66] Torrado E., Fountain J.J., Robinson R.T., Martino C.A., Pearl J.E., Rangel-Moreno J., Tighe M., Dunn R., Cooper A.M. (2013). Differential and site specific impact of b cells in the protective immune response to mycobacterium tuberculosis in the mouse. PLoS ONE.

[67] Esmail H., Lai R.P., Lesosky M., Wilkinson K.A., Graham C.M., Horswell S., Coussens A.K., Barry C.E., O’Garra A., Wilkinson R.J. (2018). Complement pathway gene activation and rising circulating immune complexes characterize early disease in hiv-associated tuberculosis. Proc. Natl. Acad. Sci. USA.

[68] Achkar J.M., Casadevall A. (2013). Antibody-mediated immunity against tuberculosis: Implications for vaccine development. Cell Host Microbe.

[69] Maekura R., Kitada S., Osada-Oka M., Tateishi Y., Ozeki Y., Fujicawa T., Miki M., Jyunnko O., Mori M., Matsumoto S. (2019). Serum antibody profiles in individuals with latent mycobacterium tuberculosis infection. Microbiol. Immunol..

[70] Hamasur B., Haile M., Pawlowski A., Schroder U., Kallenius G., Svenson S.B. (2004). A mycobacterial lipoarabinomannan specific monoclonal antibody and its f(ab’) fragment prolong survival of mice infected with mycobacterium tuberculosis. Clin. Exp. Immunol..

[71] Balu S., Reljic R., Lewis M.J., Pleass R.J., McIntosh R., van Kooten C., van Egmond M., Challacombe S., Woof J.M., Ivanyi J. (2011). A novel human iga monoclonal antibody protects against tuberculosis. J. Immunol..

[72] Zimmermann N., Thormann V., Hu B., Kohler A.B., Imai-Matsushima A., Locht C., Arnett E., Schlesinger L.S., Zoller T., Schurmann M. (2016). Human isotype-dependent inhibitory antibody responses against mycobacterium tuberculosis. EMBO Mol. Med..

[73] Lu L.L., Chung A.W., Rosebrock T.R., Ghebremichael M., Yu W.H., Grace P.S., Schoen M.K., Tafesse F., Martin C., Leung V. (2016). A functional role for antibodies in tuberculosis. Cell.

[74] Jain D., Ghosh S., Teixeira L., Mukhopadhyay S. (2017). Pathology of pulmonary tuberculosis and non-tuberculous mycobacterial lung disease: Facts, misconceptions, and practical tips for pathologists. Semin. Diagn. Pathol..

[75] Fujita J., Ohtsuki Y., Suemitsu I., Shigeto E., Yamadori I., Obayashi Y., Miyawaki H., Dobashi N., Matsushima T., Takahara J. (1999). Pathological and radiological changes in resected lung specimens in Mycobacterium avium intracellulare complex disease. Eur. Respir. J..

[76] Kunnath-Velayudhan S., Gennaro M.L. (2011). Immunodiagnosis of tuberculosis: A dynamic view of biomarker discovery. Clin. Microbiol. Rev..

[77] Schorey J.S., Sweet L. (2008). The mycobacterial glycopeptidolipids: Structure, function, and their role in pathogenesis. Glycobiology.

[78] Kitada S., Kobayashi K., Ichiyama S., Takakura S., Sakatani M., Suzuki K., Takashima T., Nagai T., Sakurabayashi I., Ito M. (2008). Serodiagnosis of mycobacterium avium-complex pulmonary disease using an enzyme immunoassay kit. Am. J. Respir. Crit. Care Med..

[79] Shibata Y., Horita N., Yamamoto M., Tsukahara T., Nagakura H., Tashiro K., Watanabe H., Nagai K., Nakashima K., Ushio R. (2016). Diagnostic test accuracy of anti-glycopeptidolipid-core iga antibodies for mycobacterium avium complex pulmonary disease: Systematic review and meta-analysis. Sci. Rep..

[80] Kitada S., Maekura R., Yoshimura K., Miki K., Miki M., Oshitani Y., Nishida K., Sawa N., Mori M., Kobayashi K. (2017). Levels of antibody against glycopeptidolipid core as a marker for monitoring treatment response in mycobacterium avium complex pulmonary disease: A prospective cohort study. J. Clin. Microbiol..

[81] Yamada K., Sugiyama T., Yasuda A., Seki Y., Hasegawa M., Hayashi Y., Tarumi O., Nakagawa T., Yamada N., Ogawa K. (2013). A study of relapse/recurrence cases after surgical treatment for patients with pulmonary nontuberculous mycobacteriosis. Kekkaku.

[82] Swenson C., Zerbe C.S., Fennelly K. (2018). Host variability in ntm disease: Implications for research needs. Front Microbiol..

[83] Maekura R., Okuda Y., Hirotani A., Kitada S., Hiraga T., Yoshimura K., Yano I., Kobayashi K., Ito M. (2005). Clinical and prognostic importance of serotyping mycobacterium avium-mycobacterium intracellulare complex isolates in human immunodeficiency virus-negative patients. J. Clin. Microbiol..

[84] Neonakis I.K., Gitti Z., Krambovitis E., Spandidos D.A. (2008). Molecular diagnostic tools in mycobacteriology. J. Microbiol. Methods.

[85] Maiden M.C., Bygraves J.A., Feil E., Morelli G., Russell J.E., Urwin R., Zhang Q., Zhou J., Zurth K., Caugant D.A. (1998). Multilocus sequence typing: A portable approach to the identification of clones within populations of pathogenic microorganisms. Proc. Natl. Acad. Sci. USA.

[86] Brown-Elliott B.A., Fritsche T.R., Olson B.J., Vasireddy S., Vasireddy R., Iakhiaeva E., Alame D., Wallace R.J., Branda J.A. (2019). Comparison of two commercial matrix-assisted laser desorption/ionization-time of flight mass spectrometry (maldi-tof ms) systems for identification of nontuberculous mycobacteria. Am. J. Clin. Pathol..

[87] Walker B.J., Abeel T., Shea T., Priest M., Abouelliel A., Sakthikumar S., Cuomo C.A., Zeng Q., Wortman J., Young S.K. (2014). Pilon: An integrated tool for comprehensive microbial variant detection and genome assembly improvement. PLoS ONE.

[88] Wood D.E., Salzberg S.L. (2014). Kraken: Ultrafast metagenomic sequence classification using exact alignments. Genome Biol..

[89] Truong D.T., Tett A., Pasolli E., Huttenhower C., Segata N. (2017). Microbial strain-level population structure and genetic diversity from metagenomes. Genome Res..

[90] Chun J., Rainey F.A. (2014). Integrating genomics into the taxonomy and systematics of the bacteria and archaea. Int. J. Syst. Evol. Microbiol..

[91] Konstantinidis K.T., Tiedje J.M. (2005). Genomic insights that advance the species definition for prokaryotes. Proc. Natl. Acad. Sci. USA.

[92] Parks D.H., Chuvochina M., Chaumeil P.A., Rinke C., Mussig A.J., Hugenholtz P. (2020). A complete domain-to-species taxonomy for bacteria and archaea. Nat. Biotechnol..

[93] Auch A.F., von Jan M., Klenk H.P., Goker M. (2010). Digital DNA-DNA hybridization for microbial species delineation by means of genome-to-genome sequence comparison. Stand. Genom. Sci..

[94] Queiroz K.D. (1998). The general lineage concept of species, species chteria, and the process of speciation and terminological recommendations. Endless Forms Species Speciation.

[95] Jolley K.A., Maiden M.C. (2010). Bigsdb: Scalable analysis of bacterial genome variation at the population level. BMC Bioinform..

[96] Matsumoto Y., Kinjo T., Motooka D., Nabeya D., Jung N., Uechi K., Horii T., Iida T., Fujita J., Nakamura S. (2019). Comprehensive subspecies identification of 175 nontuberculous mycobacteria species based on 7547 genomic profiles. Emerg. Microbes Infect..

[97] Votintseva A.A., Bradley P., Pankhurst L., del Ojo Elias C., Loose M., Nilgiriwala K., Chatterjee A., Smith E.G., Sanderson N., Walker T.M. (2017). Same-day diagnostic and surveillance data for tuberculosis via whole-genome sequencing of direct respiratory samples. J. Clin. Microbiol..

[98] Ratnatunga C.N., Lutzky V.P., Kupz A., Doolan D.L., Reid D.W., Field M., Bell S.C., Thomson R.M., Miles J.J. (2020). The Rise of Non-Tuberculosis Mycobacterial Lung Disease. Front. Immunol..

[99] Seo H., Jeon I., Kim B.S., Park M., Bae E.A., Song B., Koh C.H., Shin K.S., Kim I.K., Choi K. (2017). IL-21-mediated reversal of NK cell exhaustion facilitates anti-tumour immunity in MHC class I-deficient tumours. Nat. Commun..

